# Amsterdam Placental Workshop Group Consensus Statement definitions revisited: Basal chronic villitis

**DOI:** 10.1007/s00428-025-04152-z

**Published:** 2025-06-25

**Authors:** T.Y. Khong, C.J. Kim, B.B. Rogers

**Affiliations:** 1 Department of Anatomical Pathology, Women’s and Children’s Hospital, North Adelaide, Australia; 2 Division of Obstetrics and Gynaecology, University of Adelaide, Adelaide, SA 5005 Australia; 3 Department of Pathology, University of Ulsan College of Medicine, Asan Medical Center, Seoul, Korea; 4 Department of Pathology, Children’s Healthcare of Atlanta, Atlanta, GA 30342 USA; 5 Department of Pathology, Emory University School of Medicine, Atlanta, GA 30322 USA

**Keywords:** Definition, Nosology, Placental pathology, Chronic villitis, Villitis of unknown etiology

## Abstract

Villitis of unknown etiology (VUE) can be categorized as distal, proximal, or basal, depending on the type of chorionic villi involved. Either terminal or mature intermediate villi are affected in the distal type, while stem villi are affected in the proximal type. The Amsterdam Placental Workshop Group Consensus Statement did not amplify on the definition of basal VUE. We review the literature to explore the entity, focusing on the terminology, inflammatory infiltrate, involved structures, prevalence, and clinical significance. The prevalence of the lesion, variously defined, in placentas with differing indications for pathological examination and which could include cases with concurrent intraparenchymal VUE ranges from 6.6 to 28.3% of VUE; however, the prevalence of VUE confined only to basal and parabasal villi could be as low as 3%. We propose that the villi that are inflamed must be clearly identified as anchoring or anchored villi; that the preferred term is basal chronic villitis; and that it is diagnosed when chorionic inflammation of anchoring villi, or of villi anchored within the basal plate is seen. No studies have looked specifically at the clinical significance of basal villitis; associations with donor oocyte IVF pregnancies, fetal growth restriction, hypertensive disorders of pregnancy, pre-eclampsia, and morbidly adherent placenta have been reported by some but not confirmed by others. VUE should continue to be reported as intraparenchymal, basal, or mixed until such time that the accumulated experience of the clinical correlates suggests there is no merit in doing so.

## Introduction

Villitis of unknown etiology (VUE) is the infiltration of maternal T cells (CD8 + cytotoxic phenotype) into the fetal chorionic villi in the context of destructive villous inflammation. Prerequisite for the diagnosis of VUE is the exclusion of infectious etiologies. There are no standard protocols for specific workups for common infectious causes and workups are not being done in routine clinical practice due to relatively distinct clinicopathological features.

The VUE pattern can be categorized as distal, proximal, or basal type [1], depending on the type of chorionic villi involved. Either terminal or mature intermediate villi are affected in distal type, while stem villi are affected in proximal type. The type of chorionic villi in basal VUE has not been described. There does not seem to be any predominant pattern among the three, and mixed patterns of VUE are common [2].

The Amsterdam Placental Workshop Group Consensus Statement did not amplify on the definition of basal VUE other than the topographic location within the placenta or on its significance [3]. In this discussion paper, we review the literature and propose that the definition of basal chronic villitis needs to be explored further.

## Terminology

Terms that have been used include basal chronic villitis [4, 5], chronic basal villitis [6], basal villitis [7–10], parabasal villitis [11], basal placentitis [12], and anchoring villitis [13, 14] (Table 1).

**Table 1:** Summary of papers describing basal chronic villitis

First author [ref]	Term used for lesion	Inflammatory infiltrate	Affected villi	Clinical condition studied; prevalence
Dubruc [4]	Basal chronic villitis		Anchoring villi; basal villi along the basal plate, subjacent to the decidua	Fetal and neonatal alloimmune thrombocytopenia
Katzman [5]	Basal chronic villitis		Maternal floor villi	Hypertensive disorders of pregnancy and morbidly adherent placenta
Ernst [6]	Chronic basal villitis	Lymphocytes, histiocytes, or plasma cells	Anchoring chorionic villi	Morbidly adherent placenta
Kim [7]	Basal villitis	Mononuclear and plasma cells	Anchoring villi	Review paper
Gundogan [8]	Basal villitis		Anchoring villi embedded in the basal plate and adjacent terminal villi	Egg donor pregnancies
Redline [9]	Basal villitis		Villi adjacent to the decidua basalis	Recurrent villitis
Greer [10]	Basal villitis		Anchoring villi	Fetal growth restriction; 13.5% of VUE cases
Russell [11]	Parabasal villitis	Lymphoplasmacytic	Adjacent basal villi	Case series of VUE and correlated clinical outcome; 9.6% of VUE cases
Granat [12]	Basal placentitis			Herpetic gingivostomatitis
Labarrere [13]	Anchoring villitis	Lymphohistiocytic	Anchoring villi	Massive chronic intervillositis
Labarrere [14]	Anchoring villitis		Anchoring villi	Recurrent intrauterine fetal growth retardation
Labarrere [15]		Lymphocytes	Anchoring villi	Idiopathic small for gestational age infants
Redline [1]			Villi embedded in and adjacent to the decidua basalis	Review paper
Bang [16]	Basal VUE		Villi anchoring to basal plate	Chronic placental inflammation in twin pregnancies

where cells have no entries, no pertinent information was available in the publication

*VUE* villitis of unknown etiology

## Infiltrate

The inflammatory infiltrate has been described as being lymphocytes [15], a mixed lymphoplasmacytic infiltrate [11], mononuclear and plasma cells [7], mononuclear (lymphohistiocytic) cells [13] and lymphocytes, histiocytes, or plasma cells [6].

The supposition is that the inflammatory cells are maternal in origin. Russell describes “a mixed lymphoplasmacytic infiltrate in the maternal decidua which has ascended to involve the adjacent basal villi in the inflammatory process” [11]. Labarrere wrote that the lymphocytes were “probably of maternal origin” [15].

## Involved structures

There is general agreement that the lesion has a basal or parabasal (of the basal plate and also of the septa) distribution. There is less agreement or clarity regarding the villi that are affected, however. They have been described variously as “adjacent basal villi” [11], “villi adjacent to the decidua basalis” [9], “villi in juxtaposition to the maternal decidua” [11], “basal villi along the basal plate, subjacent to the decidua” [4], “maternal floor villi (e.g., next to the decidua basalis)” [5], “anchoring villi” or “anchoring chorionic villi” [4, 6, 7, 10, 13, 14, 16], “villi embedded in and adjacent to the decidua basalis” [1], and “anchoring villi embedded in the basal plate and adjacent terminal villi” [8].

The decidua is described as inflamed also [7, 8, 11, 12, 15].

## Prevalence

Basal villitis appears to be the least common variant of VUE.

The study by Russell probably gives a robust prevalence of the different variants of chronic villitis [11] insofar as he examined 7505 consecutively delivered singleton placentas from liveborn infants and stillborn fetuses weighing over 400 g or delivered after 20 weeks gestation. He found 55 (9.7%) had a parabasal/basal pattern, 52 (9%) had a mixed but predominantly parabasal pattern, 25 (4.3%) had a mixed but mainly focal pattern, and 443 (77%) had a totally random pattern with no basal component. Thus, villitis exclusively confined to the parabasal or basal zone constituted approximately 9.6% (55/575) of all cases of VUE.

In a study of 10,204 placentas from liveborn singletons that met obstetric and neonatal indications for pathological examination, 286 (3%) had basal villitis (defined as having lesions only in anchoring villi) and 1378 (13.5%) had chronic villitis; thus, 13.5% of VUE cases were basal in location [10].

In 19,683 placentas from high-risk pregnancies submitted from a maternal–fetal medicine practice, there were 2905 placentas with chronic villitis, of which 821 were classed as being basal. In this study, cases of basal villitis could include cases with parenchymal villitis, but all these cases were not counted as non-basal; thus, the prevalence of non-basal villitis was 71.7% [5].

Villitis showing the basal or parabasal pattern was seen in 9 (6.6%) placentas from 136 with VUE out of 1000 randomly accessioned singleton placentas from women delivered after 20 weeks gestation [17].

## Clinical significance

There are no studies that have looked specifically at the clinical significance of basal villitis, but there are some studies examining clinical conditions that have commented on the association of those clinical conditions with basal villitis.

Donor oocyte IVF pregnancies have been shown to be more likely to demonstrate VUE when compared to non-donor IVF pregnancies [18–21]. Of 33 placentas from donor oocyte IVF pregnancies, chronic deciduitis with basal villitis was found in 1 and VUE in 2, while of 60 placentas from non-donor oocyte IVF pregnancies, chronic deciduitis with basal villitis was found in 4 and VUE in 4; what is not clear is whether the 2 and 4 placentas with VUE were also those with the basal villitis [8]. Notwithstanding that, the numbers seem low to obtain statistical significance.

In a study examining whether there was a relationship between chronic villitis and fetal growth restriction, the chronic villitis was stratified based on the location of the inflammation [10]. In this study, of 10,204 placentas that were examined histologically, 286 (3%) had basal villitis, 867 (8%) had low-grade chronic villitis, and 11 (5%) had high-grade chronic villitis. Chronic villitis but not basal villitis was found to be associated with pre-eclampsia, cesarean section for non-reassuring fetal heart rate and preterm delivery. Whereas chronic villitis was associated with fetal growth restriction, basal villitis was not [10]. This was also shown in a smaller study of 63 term pregnancies by Labarrere and colleagues [15] of chronic villitis in small-for-gestational age infants. In ten women who had successive term pregnancies complicated by fetal growth restriction, basal villitis was found in seven of the first pregnancies which recurred in five; VUE was not present elsewhere in some of these placentas [14].

A large retrospective study set out to test the hypothesis that basal chronic villitis is associated with hypertensive disorders of pregnancy [5]. Both intraparenchymal chronic villitis and basal villitis, which in the study did not preclude coexisting intraparenchymal villitis, were associated with hypertensive disorders of pregnancy. The authors did find that basal villitis was more likely to occur in the presence of hypertensive disorders of pregnancy than when it was not present.

The frequency of anchoring villitis did not differ between pre-eclamptic and normotensive pregnancies across different birth weight centiles [22].

A retrospective study that aimed to describe the pathology at the implantation site in cases of morbidly adherent placenta, defined by the authors as the need for clinical intervention at delivery beyond spontaneous placental delivery or simple manual extraction of the placenta, found plasma cell deciduitis and basal chronic villitis to be highly associated with morbidly adherent placenta [6]. They did not find any increased prevalence of central chronic villitis in morbidly adherent placentas over control placentas, which were from women with a history of maternal malignancy.

In a retrospective study of recurrent villitis, two clinical patient profiles were found [9]. In one, the mothers were more likely to have histories of sexually transmitted disease, and the placentas of this group showed basal villitis. The other group of patients had diffuse VUE with attendant intervillous fibrin deposition or chronic inflammatory cells, which we interpret to be akin to villitis accompanying massive chronic intervillositis.

## Recommendations

To gain an understanding of what the clinical implications of basal chronic villitis are, it is necessary to agree on the involved structures. The next steps would be to decide whether it is pathogenically and clinically different from intraparenchymal or a mixed pattern of chronic villitis. This is important as the intents of the Amsterdam consensus were to standardize the nomenclature of placental pathology diagnosis and to make them clinically relevant.

**Fig. 1:**
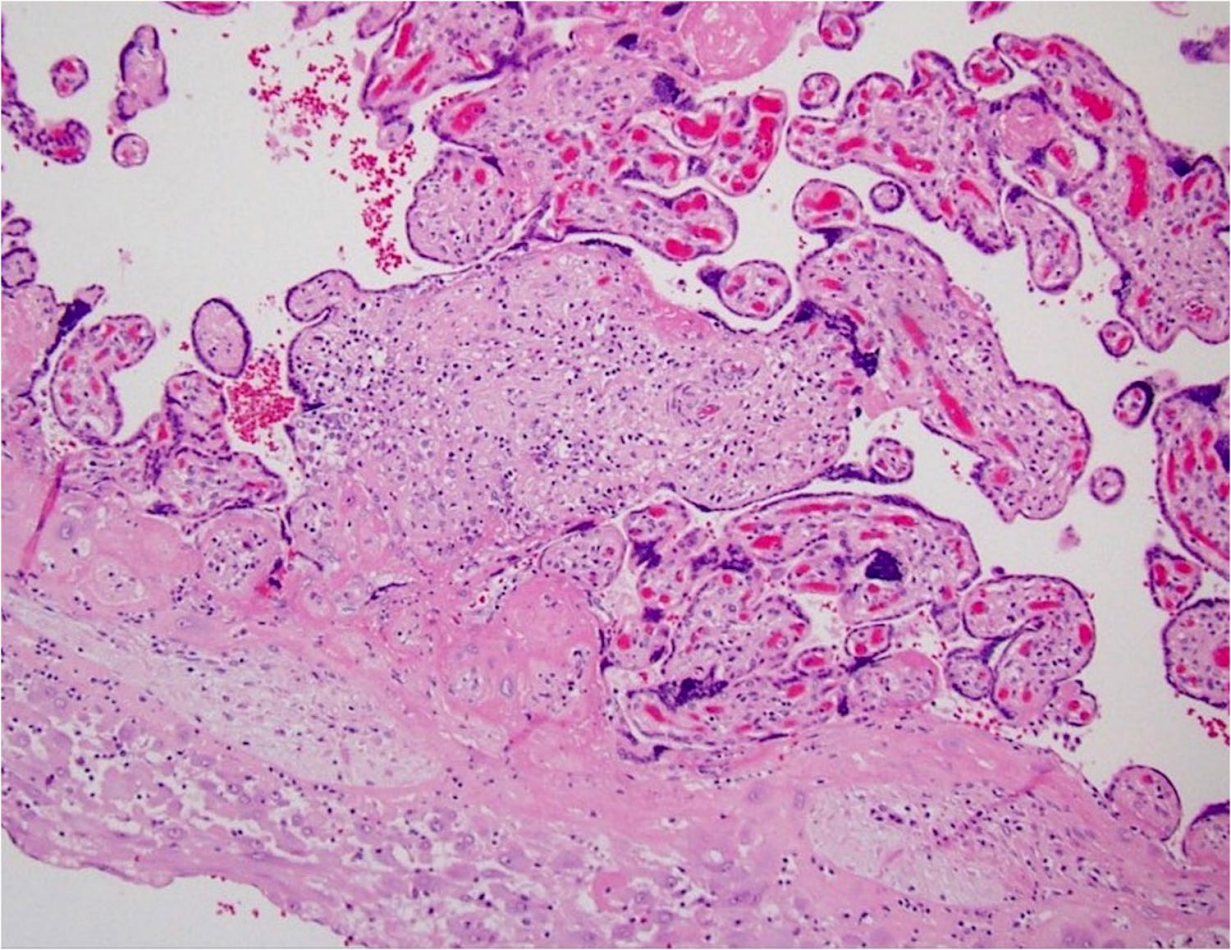
Chronic inflammation within the stroma of an anchoring villus

**Fig. 2:**
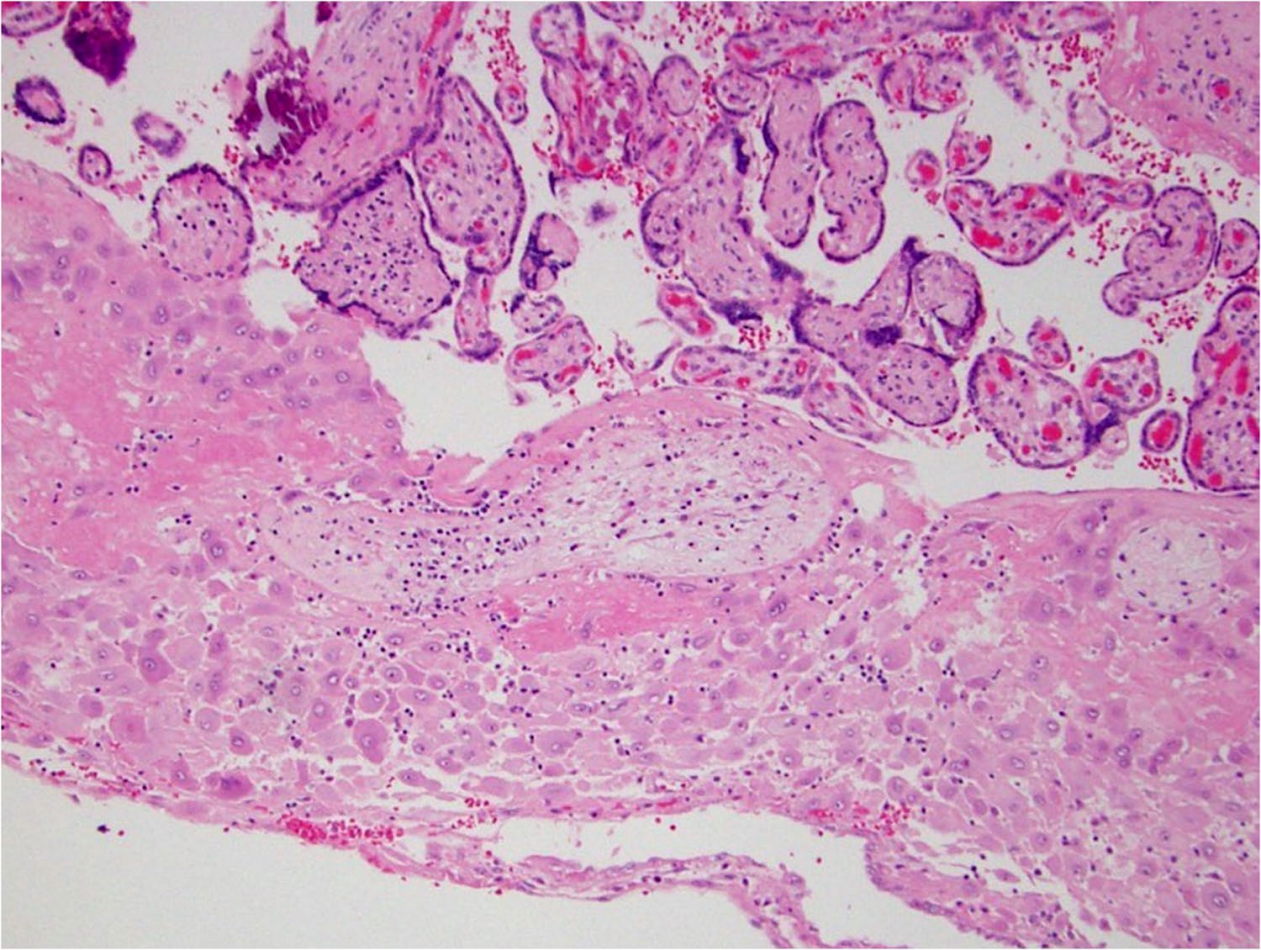
Chronic inflammation within the stroma of a villus embedded within the fibrinoid layer of the basal plate

**Fig. 3:**
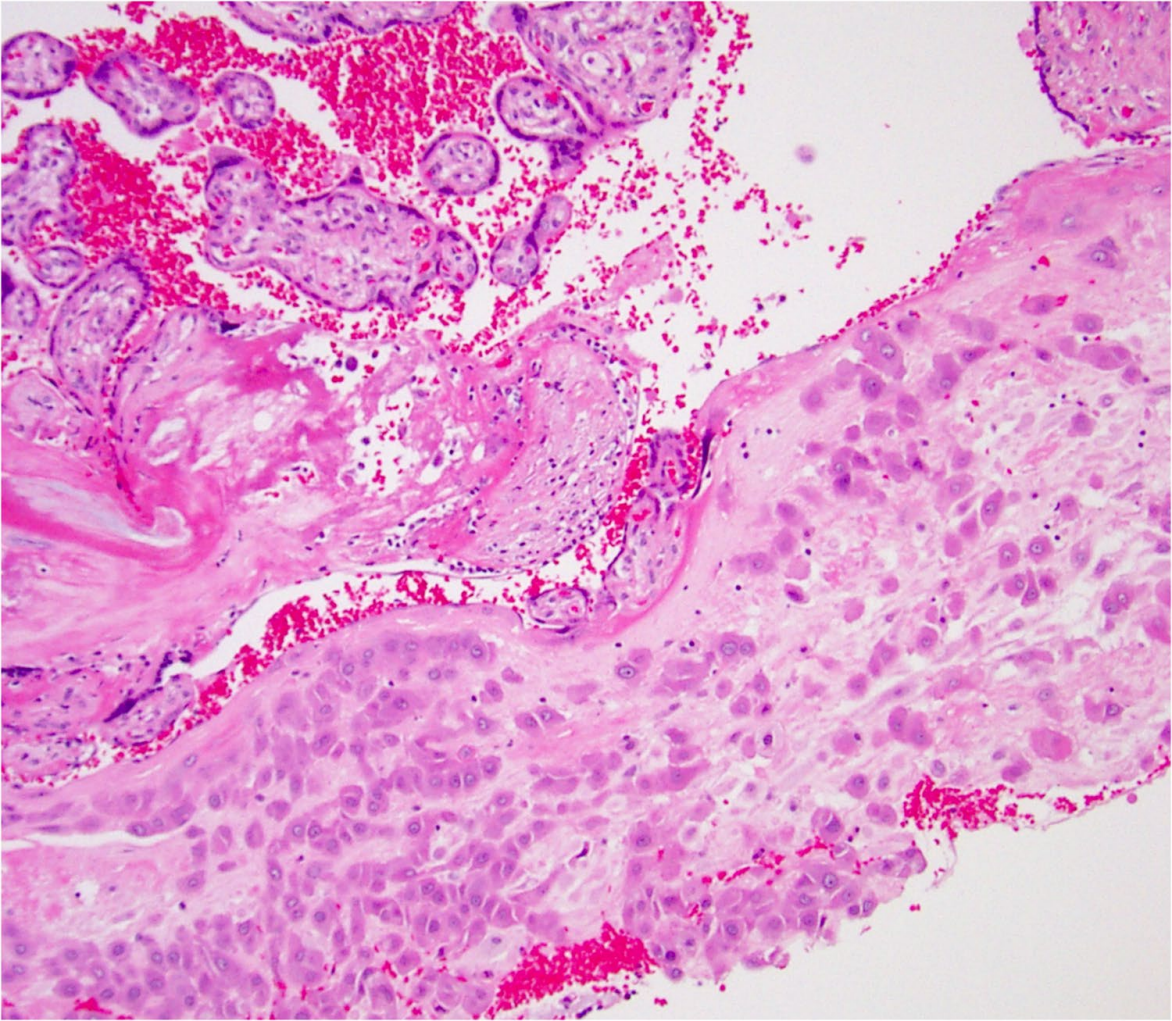
Chronic inflammation within the stroma of a parabasal villus close to the basal plate

**Fig. 4:**
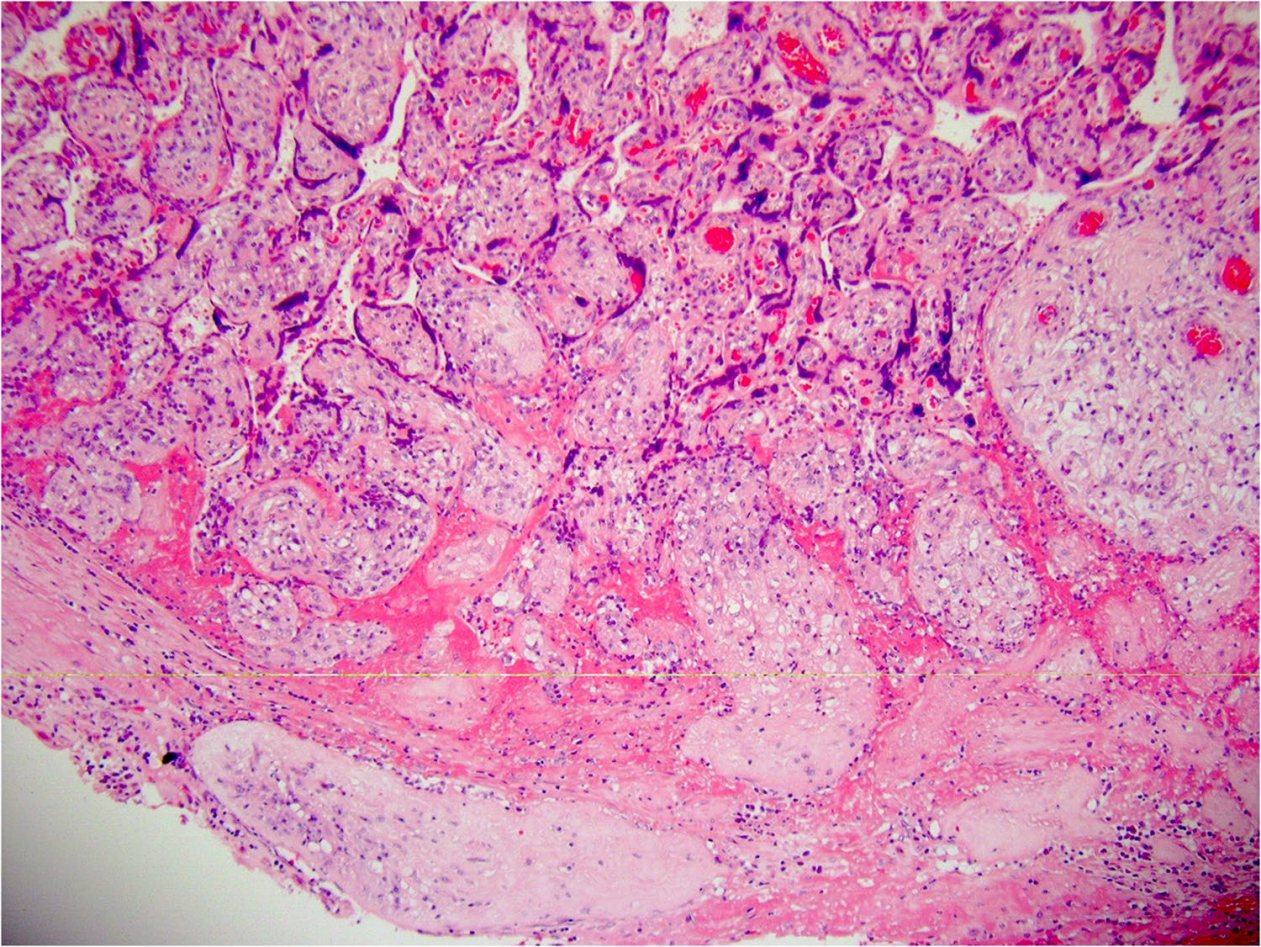
Band-like chronic inflammation of parabasal villi

**Fig. 5:**
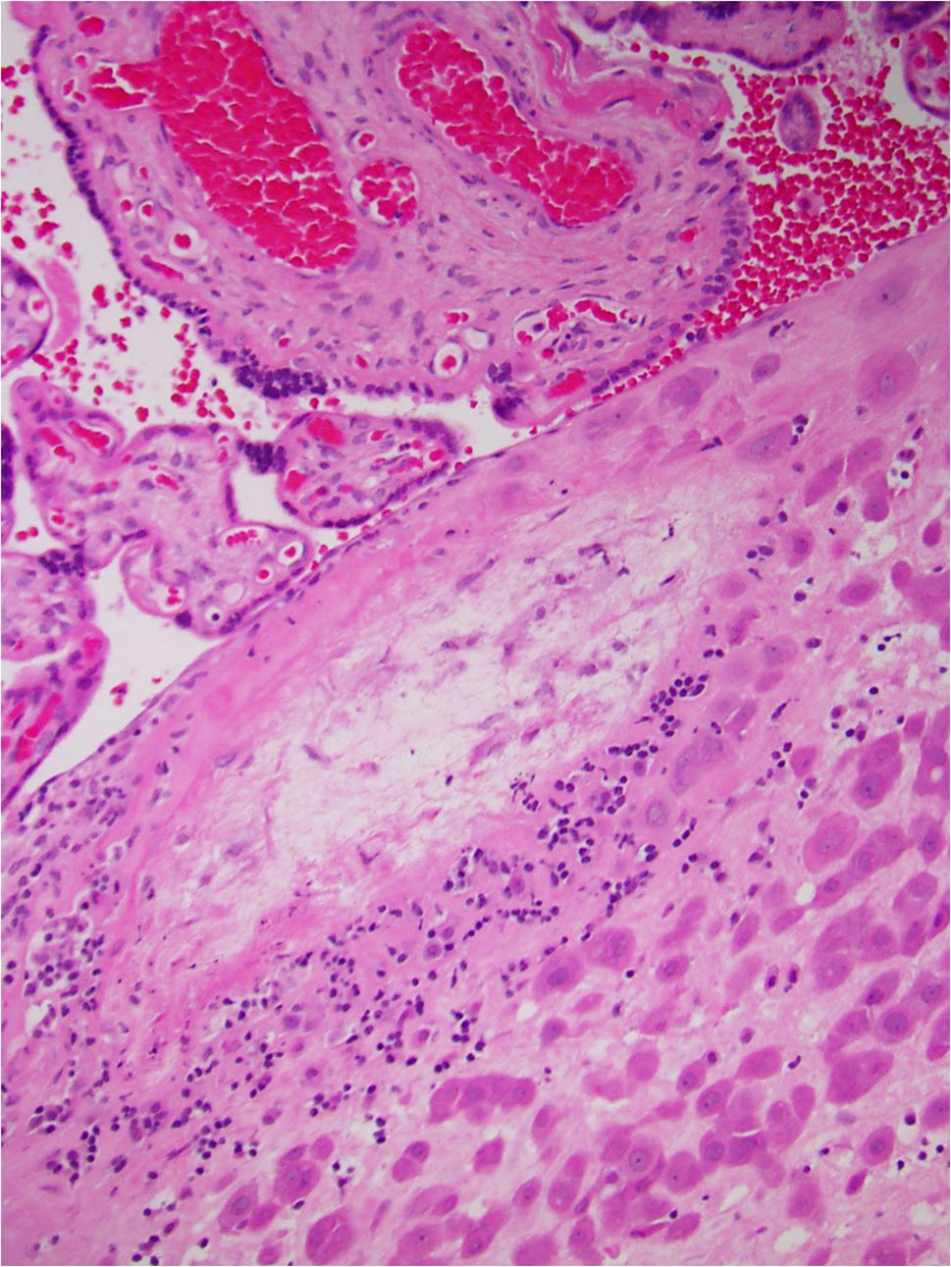
Chronic inflammation within the basal plate around a sclerosed or obsolescent anchored villus

**Fig. 6:**
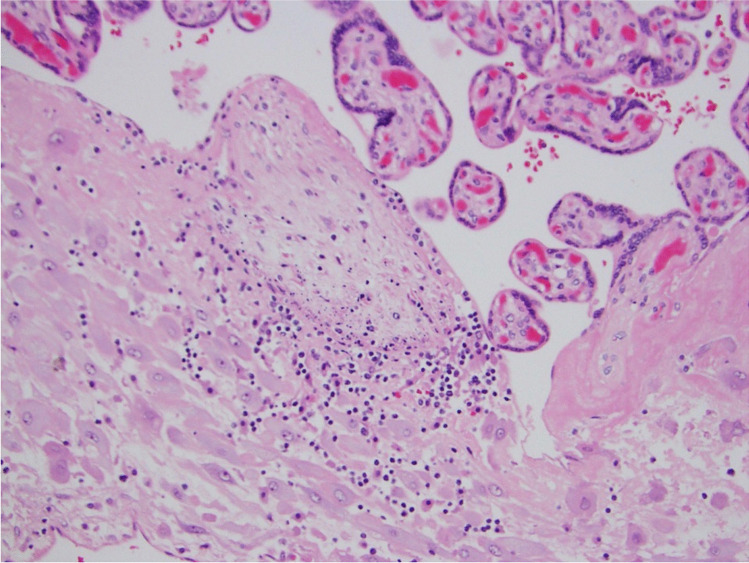
Peri-villous inflammation around an anchoring villus showing minimal intravillous inflammation

**Fig. 7:**
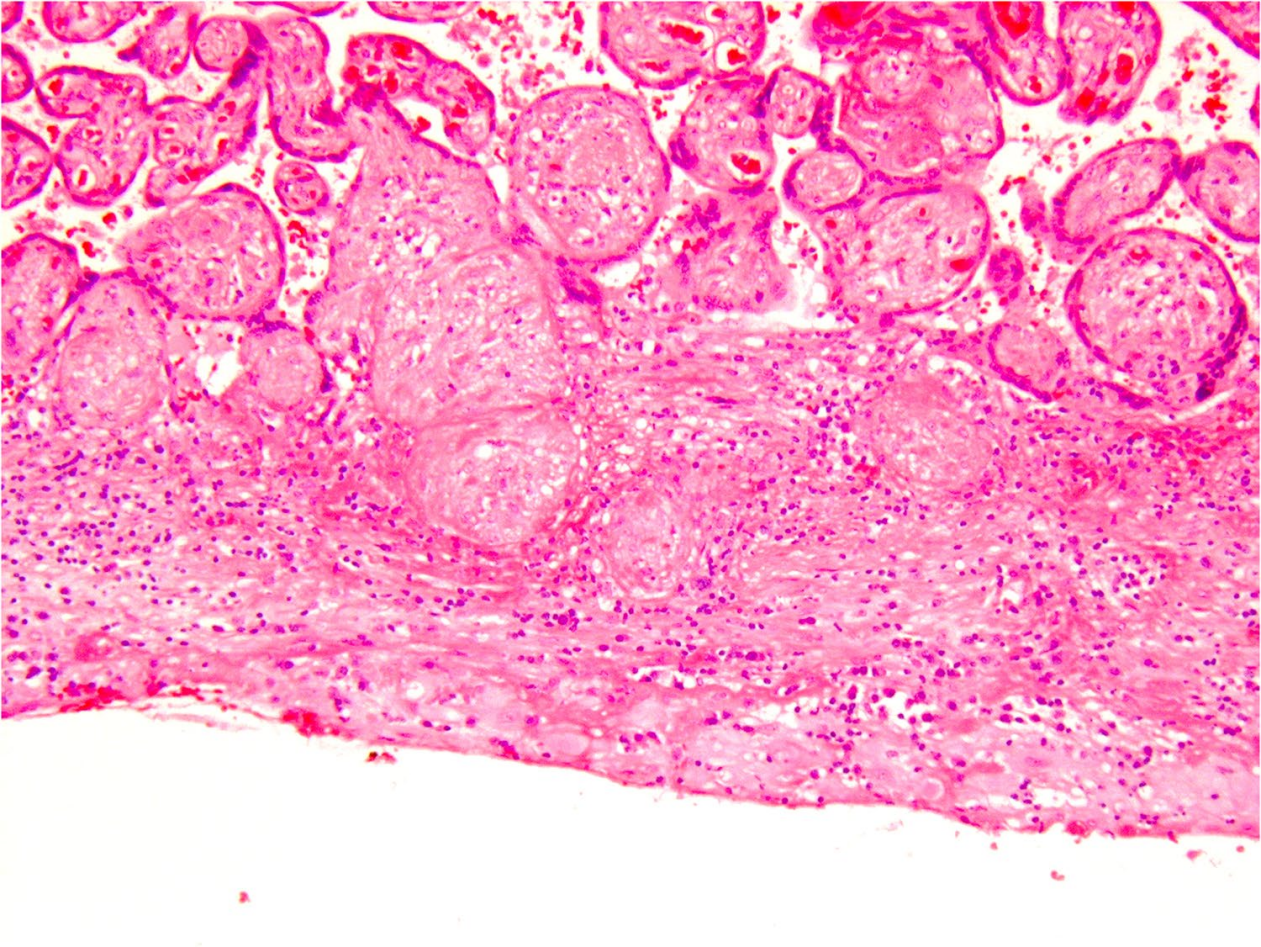
Peri-villous inflammation around anchored villi with peri-villous fibrinoid deposition

**Fig. 8:**
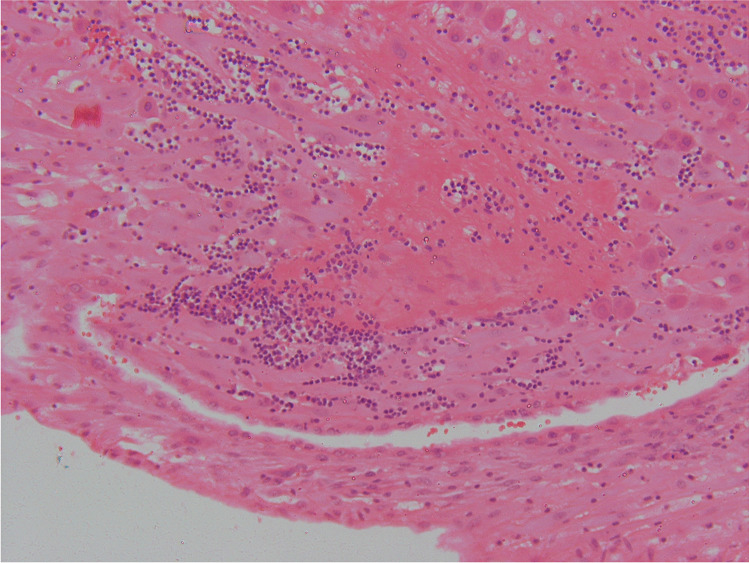
Chronic deciduitis: chronic inflammation in the basal plate absent of anchored or anchoring villi

**Fig. 9:**
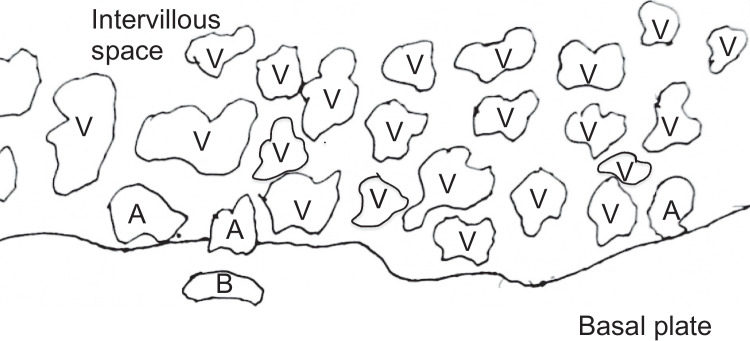
Schematic diagram illustrating different villi around the basal plate. A, anchoring villi, with differing degrees of anchoring to the basal plate; B, anchored villus within the fibrinoid of the basal plate; V, basal and parabasal villi. Note there is no demarcation or defining margin between the basal/parabasal villi and the rest of the intraparenchymal villi

We propose that the preferred term is basal chronic villitis. It is diagnosed when extant inflammation of anchoring villi or of villi anchored within the basal plate is seen (Figs. 1 and 2). The diagnosis is also accepted where the parabasal villi are not anchoring or anchored, as it is highly probable that they will be contiguous with the basal plate in a deeper serial section (Fig. 3). Inflammation of parabasal villi in a band-like manner is striking in the extent, but it is not clear if this has a different clinical implication (Fig. 4). At this stage, we do not propose a grading system, but most cases of basal villitis appear to be focal, differing from the band-like inflammation. We accept as basal chronic villitis also where there is inflammation within the basal plate around a sclerosed or obsolescent anchored villus (Fig. 5). We do not have any evidence that this represents the natural history of an inflamed basal villus, but the frequent finding of diminished intravillous inflammation with surrounding peri-villitis (Fig. 6) leads us to believe it to be so. Furthermore, the inflammation appears to be accompanied by peri-villous fibrinoid deposition in the basal plate (Fig. 7). In chronic deciduitis, fibrinoid deposition may be seen, but the inflammation is not adjacent to anchored or anchoring villi (Fig. 8). The described villi are illustrated schematically (Fig. 9).

We are cognizant of the influence of sampling on the detection of villitis and, thus, on the prevalence of basal chronic villitis. The majority of VUE can be identified by the use of four conventional placental sections [17]. The distribution of VUE is frequently non-uniform, especially for the milder cases [11], which is what basal chronic villitis appears to be. Use of en-face sections of the basal plate [23], in addition to the proposed Amsterdam workshop consensus recommendation of three perpendicular blocks of normal appearing parenchyma [3], is an additional variable.

Comparative immunophenotypic studies of the inflammatory infiltrate may provide some insight into whether basal chronic villitis is part of VUE or that intraparenchymal and basal chronic villitis are dissimilar. Most studies have not found appreciable numbers of B cells in VUE [1]. Basal chronic villitis appears to be an exception where as many as 30% of cells have been reported as being B lymphocytes; however, this was only reported as a meeting abstract [24]. Likewise, it is possible that examining the regulatory T cells and antigen presenting cell populations in the decidua may offer a clue as to whether the peri-villitis really differs from chronic deciduitis. Finally, studies demonstrating maternal origin of the inflammatory infiltrate in VUE [25–28] have investigated only intraparenchymal VUE but not basal chronic villitis or a mixed pattern VUE, and this may be worthy of further study.

We also propose that VUE should continue to be reported as intraparenchymal, basal, or mixed, until such time that the accumulated experience of the clinical correlates suggests there is no merit in doing so. Parenthetically, while intraparenchymal VUE is categorized as being distal or proximal based on the type of chorionic villi involved [1], histological classification of villous types (which are named by their expected position in villous trees as terminal, intermediate, or stem villi) is difficult and unreliable [29] and can be compounded by villous maturational defects [30]. It is possible then that there may be only two categories—intraparenchymal and basal.
